# From the Bio-Psycho-Social Model to the Development of a Clinical–Forensic Assessment Tool for Chronic Pain in Victims of Violence: A Research Protocol

**DOI:** 10.3390/brainsci14100953

**Published:** 2024-09-24

**Authors:** Allison Uvelli, Erica Pugliese, Alessandra Masti, Giulia Salvo, Cristina Duranti, Giacomo Gualtieri, Fabio Ferretti

**Affiliations:** 1 Department of Medical Science, Surgery and Neurosciences, University of Siena, 53100 Siena, Italy; alessandra.masti@unisi.it (A.M.); ferrefa@unisi.it (F.F.); 2 Department of Clinical Psychology, University of Amsterdam, 1012 Amsterdam, The Netherlands; e.pugliese@uva.nl

**Keywords:** violence against women, chronic pain, bio-psycho-social model, clinical-forensic assessment tool

## Abstract

Violence against women impacts a minimum of 35% of the global female population, encompassing sexual, physical, and psychological forms. Perpetrators of this violence include partners, family members, or strangers. Its ramifications are substantial, evident in the prevalence of chronic pain reported by between 48% and 84% of women who have experienced abuse, with an odds ratio of 2.08. Notably associated diagnoses include pelvic/vaginal pain, fibromyalgia, irritable bowel syndrome/bowel symptoms, abdominal pain, migraine/headache, and back and neck pain. These diagnoses significantly limit a woman’s ability to participate in daily activities, such as exercising or working, leading to genuine disability. Despite substantial evidence, the precise cause and etiology of these conditions remain unclear. Adhering to the bio-psycho-social model, it is conceivable that chronic pain in victims of violence cannot be attributed to a single factor alone, but rather to a combination of all three: biological, psychological, and social factors. Uncovering these factors could have significant clinical and legal implications. On one hand, it would be possible to conduct screenings to avoid developing chronic pain. and guide individuals toward the correct treatment. On the other hand, victims could seek compensation for chronic pain resulting from violence. Considering the limited knowledge about the causes of chronic pain and the absence of tools to identify risk factors or a set of tests for evaluating victims of violence, the goal of the research described in this project protocol is to pinpoint the specific contributing factors for chronic pain due to violence victimization. Additionally, it aims to devise a comprehensive protocol for assessing these factors in forensic science.

## 1. Introduction

A significant amount of literature highlights the high prevalence of chronic pain among individuals who have been subjected to violence [1,2,3]. This is documented in 48% [4] to 84% [5] of abused women, with an odds ratio of 2.08 [6]. Exposure to trauma in general increases the risk of developing chronic pain [7,8]. Chronic pain is described as long-lasting pain for at least three months, adversely affecting the functioning or well-being of the individual [9]. Pain is defined as “an unpleasant sensory and emotional experience associated with, or resembling that associated with, actual or potential tissue damage” [10]. Many studies focus on different forms of chronic pain, such as pelvic/vaginal pain [11], fibromyalgia [12], irritable bowel syndrome/bowel symptoms [13], abdominal pain [14], migraine/headache [15], back pain [16], and neck pain [17], all of which tend to become chronic conditions. Among individuals affected by chronic pain, those with a background of trauma report higher levels of pain intensity [18,19], increased affective distress, and higher disability [20,21] than those without a trauma history. The exact cause and etiology of these conditions are still unclear despite the evidence. It is hypothesized that this pain may have psychological components that are not objectively detectable, described as nociplastic pain [22], which results from changes in the function of pain-related sensory pathways in the periphery and the peripheral nervous system, leading to heightened sensitivity [23]. This is supported by the painful areas not corresponding to the injured areas [24], as well as by the absence of pathognomonic clinical findings/biomarkers, the presence of subjective symptoms, and the difficulty of describing them [25]. Given the intricacy of the current situation, the bio-psycho-social model [26] could provide a clearer insight into the underlying causes. According to this approach, disease results from a mix of biological, psychological, and social dimensions interacting differently for each individual. By using this model, we can understand that chronic pain in victims of violence is not caused solely by a single factor, whether psychological, sociological, or biological, but rather by an interplay of all three. A recent scoping review [27] attempts to explain the most influential risk factors for the development of chronic pain in violence victims, confirming that all three variables are present. These are subdivided into fourteen categories: nine biological, and five psycho-social. The biological one includes weight conditions, acute upper/lower respiratory tract infection, genitourinary conditions, cardiovascular symptoms and conditions, endocrine disease, hormonal conditions, gastrointestinal disorders, skin problems, and specific inflammations. The psycho-social category includes mental health disorders, the use of psychoactive substances, life events, life quality, and personal characteristics. Within these 14 categories, there are numerous indicators, symptoms, and conditions, totaling 65. Unfortunately, the way they interact with each other and which ones are involved in the development of disease is not yet fully established. This condition has many implications. First of all, clarifying which risk factors induce the onset of chronic pain allows for prevention, screening, and proper treatment for women. If patients show major risk factors, preventive measures can be implemented to address the development and/or persistence of pain. This could lead to the development of new solutions and therapeutic strategies from a clinical perspective, as well as guidance on creating new tools to assess the presence of these factors. Individualized treatment is currently not possible because the specific significant variables are unknown, as well as the cause–effect association. Then, there are forensic implications to consider when evaluating damage caused by violence, as chronic pain resulting from such incidents can have invalidity consequences. In this context, it is necessary to use valid and reliable tools for verifying the presence of risk factors and diagnosing this condition, which currently do not exist. The research described in this study protocol aims to understand the causes of chronic pain in abused women and to measure the differences in psychological and legal aspects between abused women with chronic pain and those without. After completing these two assessments, it will be possible to identify the type of pain and guide these women toward appropriate, individualized treatment in line with precision medicine. Furthermore, this will assist us in seeking compensation for the injuries they have suffered. The study’s novelty lies in clarifying the etiology of pain, leading to improved diagnosis and personalized treatment, and quantifying the disability caused by chronic pain in medico-legal terms.

### Study Objectives

The primary goal of the study outlined in this protocol is to validate a set of medico-legal tools, such as scales and etiological factors, to differentiate between the biological, psychological, and social aspects of pain in women who have been victims of violence. The secondary objective of the study is to create a method to assist professionals in guiding women with chronic pain towards personalized treatment based on the results of these tools. Additionally, the evaluation procedures in this study will provide an objective assessment that can be used to substantiate claims for compensation due to physical or psychological harm resulting from violence, which is currently not definitively recognized.

## 2. Materials and Methods

### 2.1. Participants: Inclusion and Exclusion Criteria

Patients with chronic pain admitted to University Hospital S. Maria alle Scotte will be considered for inclusion, and screened for eligibility. Victims admitted to Italian Anti-Violence Centers will be considered for inclusion, and screened for eligibility. The general population will have access through flyers hanging inside the hospital and will be screened for eligibility. Eligible participants will receive a letter with an invitation to participate in the study. If participants do not respond to the letter within seven days, project members will call them to ask if they are interested in participating. The inclusion criteria require women to be at least 18 years old and to speak Italian, with or without chronic pain. Exclusion criteria include being male, under 18 years old, a non-Italian speaker, and having cognitive deficits.

### 2.2. Study Setting

The study will be carried out in collaboration with the University Hospital S. Maria alle Scotte (Siena, Italy), Italian Anti-Violence Centers, and the general population. Recruitment will commence in two months with the assistance of these services. Evaluation of the victims will be conducted based on the women’s needs either at the hospital or at the anti-violence centers. The general population will be enrolled through an opportunistic sampling. The project will last three years.

### 2.3. First Step: Analyzing the Etiology

For the first phase of the project, we plan to analyze the causes of chronic pain using a case–control study design. We will select two groups of women—one with chronic pain (cases) and the other without (controls). The two groups will be matched based on age and education level. We will investigate the exposure to psychosocial and biological factors to understand the causes of chronic pain. The sample size is calculated according to the quality of OR estimation: considering a relative precision of 0.5, a level of confidence of 95%, an expected prevalence of the outcome in the absence group of 0.15, and an expected OR of 7, the sample size is determined as 96 women (48 in each group).

#### 2.3.1. Measures

A checklist, based on the scoping review by Uvelli et al. [27], was created to assess risk and protective factors (Table 1). The only demographic information that will be collected is age and education level.

#### 2.3.2. Statistical Analysis

Data collected in this research step will be analyzed using odds ratios (with their 95% confidence intervals), binary logistic regression models to assess the weight of the risk factors, and Rasch analysis to validate the checklist. The analysis will be performed with Jamovi software (https://www.jamovi.org/, accessed on 31 July 2024).

### 2.4. Second Step: Building an Assessment Battery of Tools

During the second step, a group of women victims of violence will be enrolled, and a psycho-diagnostic evaluation of this sample will be performed to determine the psychological differences related to chronic pain. Starting from the existing literature and/or from the validated scales traditionally used to assess impairments related to psychological or biological pain, a new measure will be developed and will be tested among the women victims of violence. This second step aims to develop a psychometric measure useful for discriminating between psycho-social and biological aspects of pain. Following the guidelines about the sample size for validation studies (10–15 subjects for each item in the scale), and using the total scores of each test as if they were individual items (max 15), a group of about 100 women victims of violence will be enrolled for the second step of the research. An additional sample of women will be selected to complete questionnaires if the established number is not reached, to account for possible dropouts.

#### 2.4.1. Measures

Major clinical and forensic tests will be used to evaluate the following:

##### Life Story

The Childhood Trauma Questionnaire—Short Form (CTQ-SF) is a questionnaire designed to evaluate childhood maltreatment in individuals [28]. The questionnaire consists of 28 items grouped into five categories: physical abuse, emotional abuse, sexual abuse, physical neglect, and emotional neglect. Participants rated each item on a 5-point Likert scale, with higher scores indicating a more severe experience of childhood maltreatment. The cutoff scores for each category are as follows: ≥10 for physical abuse; ≥13 for emotional abuse; ≥8 for sexual abuse; ≥10 for physical neglect; and ≥15 for emotional neglect [29]. This questionnaire also demonstrates strong internal consistency, with a Cronbach’s α score of 0.88, and in a sample of victims, an alpha between 0.81 and 0.88 [30].

The Young Schema Questionnaire–Short Form (YSQ–SF) [31] is a survey with 90 items where respondents rate their level of agreement with statements on a 6-point Likert scale ranging from 1 (completely untrue of me) to 6 (describes me perfectly). Each statement corresponds to one of the 18 schemas outlined by Young [32]. Responses are then categorized into one of the 18 schemas and further grouped into one of five schema domains. The YSQ–SF has demonstrated adequate test–retest reliability (rs = 0.50–0.82) and internal consistency (α = 0.83–0.96) in various samples, including undergraduate and adult populations [33] and victims of violence [34].

The Attachment Style Questionnaire (ASQ) [35,36] is a questionnaire consisting of 40 items, designed for young adults and individuals with or without experience in romantic relationships. The items are presented as statements, and participants respond using a 6-point Likert scale ranging from ‘totally disagree’ to ‘totally agree’. The items are categorized into five subscales: confidence (in self and others) (CON), discomfort with closeness (DWC), relationships as secondary compared with achievements (RAS), need for approval related to fear of rejection (NFA), and preoccupation with relationships (PWR). Subscale scores are calculated as the mean of item responses within each subscale. The ASQ has demonstrated adequate test–retest reliability (rs = 0.67–0.80) and internal consistency (α = 0.76–0.85) in victims [37].

##### Psychological Symptoms

The Minnesota Multiphasic Personality Inventory-2 (MMPI-2) [38] is the most used personality questionnaire for evaluating personality profiles and psychopathology. The questionnaire comprises 567 items with “true” or “false” response options. The MMPI-2 includes 10 clinical scales that assess the significant dimensions of personality, such as Hypochondriasis, Depression, Hysteria, Psychopathic Deviate, Masculinity–Femininity, Paranoia, Psychasthenia, Schizophrenia, Hypomania, and Social Introversion. Additionally, it has 8 validity scales, 15 content scales, and 15 supplemental scales, which provide useful information about specific symptoms and different personality variables. Scale scores are calculated using standardized T scores, and a score of >65 indicates the presence of significant psychological problems [39]. This questionnaire is commonly used in forensic settings, particularly those related to violence [40].

The Symptom Checklist 90 Items Revised (SCL-90-R) [41] is a self-report tool that evaluates subjective impairment caused by physical and psychological symptoms. It comprises 90 items grouped into nine symptom scales: somatization, obsessiveness, insecurity in social contact, depressiveness, anxiety, aggressiveness/hostility, phobic anxiety, paranoid thinking, and psychoticism. The internal consistencies for the scales of the SCL-90-R ranged from α = 0.76 to α = 0.92, with the global characteristic score at α = 0.97 for adults, and 0.96 for victims [42].

The Beck Depression Inventory (BDI-II) [43] is used to evaluate depressive symptoms in a clinical and research setting. It has 21 items, each rated from zero (no symptom) to three (severe symptom). The areas covered include sadness, pessimism, feelings of failure, loss of joy, guilt, punishment, self-rejection, self-reproach, suicidal thoughts, crying, restlessness, loss of interest, inability to make decisions, worthlessness, loss of energy, change in sleeping habits, irritability, change in appetite, difficulty concentrating, fatigue, and loss of sexual interest. The BDI-II exhibits an internal consistency of 0.81. The scoring ranges are as follows [44]: <12: no depression, or clinically unremarkable or remitted; 12–19: mild depressive syndrome; 20–28: moderate depressive syndrome; ≥29: major depressive syndrome.

The State-Trait Anxiety Inventory-Y (STAI-Y) [45] determines the presence of anxious symptoms. It has two sub-scales, each with 20 items: state anxiety (S-STAI-Y), which measures the present level of anxiety, and trait anxiety (T-STAI-Y), which assesses the habitual level of anxiety in everyday life. Both questionnaires use a scoring system from zero to four, with higher scores indicating higher levels of anxious symptoms (no or low anxiety, 20–40; mild anxiety, 40–50; moderate anxiety, 50–60; high anxiety, >60). The S-STAI-Y showed an internal consistency of 0.80, as did the T-STAI-Y.

The Posttraumatic Stress Disorder Checklist (PCL-5) [46] is used to assess PTSD for various traumatic experiences. The PCL-5 consists of 20 items that correspond to the DSM four-factor conceptualization of PTSD and its symptom clusters: intrusion symptoms (items 1–5), avoidance symptoms (items 6–7), negative changes in cognition and mood (items 8–14), and increased arousal and reactivity (items 15–20). The PCL-5 is divided into four subscales corresponding to the four mentioned symptom clusters. Each item is rated on a 5-point Likert-type scale from 0 (not at all) to 4 (extremely). The PCL-5 provides a total score ranging from 0 to 80, with higher scores indicating a PTSD symptoms related to the event. The PCL-5 refers to the past 30 days. The α in a sample of victims was 0.96 [47].

##### Pain and Quality of Life

The Toronto Alexithymia Scale-20 (TAS-20) [48] is a self-report questionnaire in which participants rate their agreement with statements on a 5-point Likert scale. This yields a total score as well as subscale scores for difficulty identifying feelings (DIF), difficulty describing feelings (DDF), and externally oriented thinking (EOT). The highest possible score on the TAS-20 is 100. Total TAS-20 scores of 51 or higher are considered clinically significant, with scores between 52 and 60 indicating borderline alexithymia and scores of 61 or higher representing alexithymia. The reliability of the total score was measured using Cronbach’s α, which was found to be 0.89, and for DIF, DDF, and EOT specifically, the values were 0.85, 0.88, and 0.70, respectively [49].

The Illness Behavior Questionnaire (IBQ) [50] is a self-report tool of 62 items used to measure illness behavior. Respondents use a yes/no format to express whether the question represents them or not, with ‘abnormal’ behaviors being scored one point. It comprises seven subscales: General Hypochondriasis (anxious health-related concern), Disease Conviction (belief that a real disease is present), Psychological vs. Somatic Functioning (tendency to somaticize), Denial (tendency to attribute life stress to physical problems), Affective Inhibition (inability to express personal feelings to others), Affective Disturbance (anxiety, depression), and Irritability (anger, friction).

The Modified Somatic Perception Questionnaire (MSPQ) [51] is divided into 13 items designed to assess somatic complaints and autonomic perception in patients experiencing pain. Patients rate the extent to which they have been bothered by each symptom/item on a 4-point Likert scale, with 0 indicating “not at all” and 3 indicating “extremely bothered”. Higher scores indicate a greater degree of somatic complaints. The questionnaire has shown to have an internal consistency of 0.77.

The West Haven-Yale Multidimensional Pain Inventory (WHYMPI) [52] is an inventory comprising 52 items, three parts, and several subscales. The first part assesses five dimensions of the pain experience: perceived interference of pain in everyday functioning, support and concern of significant others, pain severity, self-control, and negative mood. Part II explores the responses of significant others to communications of pain with three subscales: the perceived frequency of punishing, solicitous, and distracting responses. The third part analyzes the patients’ report of their participation in four categories of frequent daily activities: household chores, outdoor work, activities away from home, and social activities. The reliability estimates for all scales appear to be quite satisfactory, ranging from 0.70 to 0.90.

The Short Form 36 Health Survey Questionnaire (SF-36) [53] contains 36 items divided into eight domains. These domains are as follows: physical functioning (10 items), social functioning (2 items), role limitations due to physical problems (4 items), role limitations due to emotional problems (3 items), mental health (5 items), vitality/energy (4 items), bodily pain (2 items), and general health perceptions (5 items). Each domain has a subscale score ranging from 0 to 100, with higher scores indicating better well-being in that particular domain.

#### 2.4.2. Statistical Analysis

**Figure 1:**
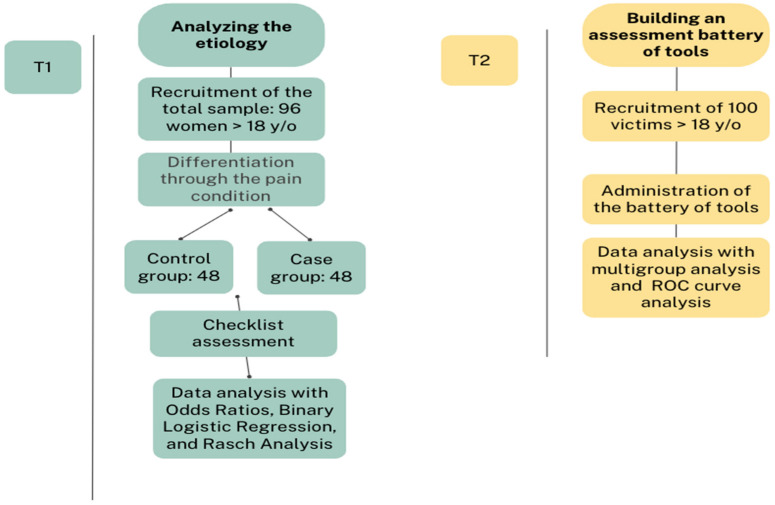
Overview of the study design.

This specific set of tools will be validated using structural equation models, considering only the total score of each questionnaire. This will help establish how this type of pain is defined by the dimension investigated through the used tools. A multigroup analysis will be conducted to determine which questionnaire is better suited for the sample and for the aim of the project. Through ROC curve analysis, it will be possible to distinguish between cases with pain and those without pain. At the end of this process, it will be possible to establish a total diagnostic cut-off. The analysis will be performed with Jamovi software. (See the following flowchart to understand the different phases of the project, Figure 1).

## 3. Discussion

This study has the potential to significantly influence both future research and clinical practice in the fields of IPV, physical health, and mental health of victims. Its importance and applicability extend well beyond the academic sphere, especially in the domains of clinical and forensic support. From a clinical perspective, the research highlights the crucial need for healthcare professionals considering the possibility of past violence when treating women with chronic pain. This awareness can lead to more accurate diagnoses and tailored treatment plans. Conversely, professionals working in anti-violence organizations should recognize that chronic pain may be a symptom of past violence, which could influence their treatment approach. Effective treatment for women who have experienced violence will benefit from a collaborative, integrated approach between healthcare professionals and anti-violence professionals. This collaboration ensures that both the physical and psychological aspects of treatment are comprehensively addressed. Incorporating this understanding into health policies, guidelines, and prevention practices is essential. It can guide victims to personalized bio-psycho-social care that improves outcomes and provides more targeted support. Screening practices, such as assessing chronic pain in women and identifying potential experiences of violence exposure, will enhance the ability to intervene early and provide appropriate care. From a forensic perspective, the results of the study are also of great importance. Psychologists in multidisciplinary teams will be better equipped to assess the psychological impact of violence and its relationship to chronic pain. This will improve forensic assessments and help to make more informed compensation claims by considering chronic pain as a consequence of violence. The use of “life-story questionnaires” in this project introduces innovative methods for understanding re-victimization [54,55], reasons for remaining in abusive relationships [56,57], and their impact on chronic pain development [58,59]. Additionally, psychological symptom questionnaires and pain and quality of life assessments are crucial for both diagnosing and understanding the broader implications of violence on health [60,61,62,63]. Overall, this project not only advances forensic assessment but also sets a precedent for future research, emphasizing the importance of an integrated approach to handling the complex interactions between violence, chronic pain, and psychological well-being [64,65].

## Figures and Tables

**Table 1:** Checklist in Italian, English, and Spanish (for English or Spanish validation contact the corresponding author).

Categorie BIO-PSICO-SOCIALI—BIO-PSYCHO-SOCIAL Categories—Categorías BIO-PSICO-SOCIAL	SI/YES	NO
Sovrappeso/obesità—Overweight/obesity—Sobrepeso/obesidad		
Sottopeso—Underweight—Bajo peso		
Infezioni delle vie respiratorie ricorrenti (sinusiti, congestioni nasali)—Recurrent respiratory infections (sinusitis, nasal congestion)—Infecciones recurrentes del tracto respiratorio (sinusitis, congestión nasal)		
Allergie che comportano sintomi respiratori (asma, rinite allergica)—Allergies resulting in respiratory symptoms (asthma, allergic rhinitis)—Alergias que provoquen síntomas respiratorios (asma, rinitis alérgica)		
Infezioni sessualmente trasmissibili (AIDS, clamidia)—Transmissible sexual infections (AIDS, chlamydia)—Infecciones de transmisión sexual (SIDA, clamidia)		
Infezioni delle vie urinarie ricorrenti (cistiti, uretriti, pielonefriti)—Recurrent urinary infections (cystitis, urethritis, pyelonephritis)—Infecciones recurrentes del tracto urinario (cistitis, uretritis, pielonefritis)		
Infezioni ginecologiche ricorrenti (vaginiti, vulviti, cerviciti)—Recurrent gynecological infections (vaginitis, vulvitis, cervicitis)—Infecciones ginecológicas recurrentes (vaginitis, vulvitis, cervicitis)		
Rigonfiamento vaginale—Vaginal swelling—Hinchazón vaginal		
Perdite urinarie ricorrenti—Recurrent urine leakage—Fugas urinarias recurrentes		
Vescicole/ulcere genitali—Genital vesicles/ulcers—Vesículas/úlceras genitales		
Sanguinamento ricorrente dopo i rapporti sessuali—Recurrent bleeding after sexual intercourse—Sangrado recurrente después de las relaciones sexuales		
Prolasso uterovaginale—Uterovaginal prolapse—Prolapso uterovaginal		
Ipertensione arteriosa (pressione alta)—Arterial hypertension (high blood pressure)—Hipertensión arterial (presión arterial alta)		
Palpitazioni cardiache—Heart palpitations—Palpitaciones del corazón		
Disturbo del metabolismo dei lipidi—Lipid metabolism disorder—Trastorno del metabolismo de los lípidos		
Diabete—Diabetes—Diabetes		
Ipercolesterolemia—Hypercholesterolemia—Hipercolesterolemia		
Patologie tiroidee (ipertiroidismo, ipotiroidismo)—Thyroid pathologies (hyperthyroidism, hypothyroidism)—Patologías tiroideas (hipertiroidismo, hipotiroidismo)		
Sintomi della menopausa intensi (vampate di calore, sudorazione, secchezza vaginale, osteoporosi)—Intense menopause symptoms (hot flushes, sweating, vaginal dryness, osteoporosis)—Síntomas intensos de menopausia (sofocos, sudoración, sequedad vaginal, osteoporosis)		
Ciclo mestruale irregolare/assente—Irregular/absent menstrual cycle—Ciclo menstrual irregular/sin período		
Reflusso gastro-esofageo—Gastroesophageal reflux—Reflujo gastroesofágico		
Alterazioni ricorrenti nel transito intestinale (stitichezza, costipazione, diarrea)—Recurrent alterations of intestinal transit (constipation, diarrhea)—Alteraciones recurrentes en el tránsito intestinal (estreñimiento, diarrea)		
Problemi/irritazioni cutanee (dermatite, eczema, rash)—Skin problems/irritations (dermatitis, eczema, rash)—Problemas/irritaciones de la piel (dermatitis, eczema, erupción)		
Otiti/congiuntiviti ricorrenti—Recurrent otitis/conjunctivitis—Infecciones de oído/conjuntivitis recurrentes		
Infiammazione muscolare—Muscle inflammation—Inflamación muscular		
Osteoartrite—Osteoarthritis—Osteoartritis		
Disturbi del sonno (insonnia, ipersonnia)—Sleep disorders (insomnia, hypersomnia)—Trastornos del sueño (insomnio, hipersomnia)		
Disturbo d’ansia (disturbo di panico, disturbo di ansia sociale, disturbo d’ansia generalizzato, fobia specifica)—Anxiety disorder (panic disorder, social anxiety disorder, generalized anxiety disorder, specific phobia)—Trastorno d’ansiedad (trastorno de pánico, trastorno de ansiedad social, d’ansiedad generalizada, fobia específica)		
Disturbo dell’umore (depressione, disturbo bipolare)—Mood disorder (depression, bipolar disorder)—Perturbación de’estado de ánimo (depresión, trastorno bipolar)		
Disturbo da stress post-traumatico—Post-traumatic stress disorder—Trastorno de estrés postraumático		
Disturbo somatico (sintomi somatici, ipocondria, disturbo di conversione)—Somatic disorder (somatic symptoms, hypochondria, conversion disorder)—Trastorno somático (síntomas somáticos, hipocondría, trastorno de conversión)		
Richiesta di parere medico per un disturbo senza poi ricevere assistenza per quello—Request for medical advice for a disorder without receiving assistance for it—Solicitar consejo médico para un trastorno sin recibir luego asistencia para ello		
Disturbo alimentare (anoressia, bulimia, binge eating)—Eating disorder (anorexia, bulimia, binge eating)—Trastorno alimentario (anorexia, bulimia, atracones)		
Diagnosi di qualsiasi disturbo psicologico—Diagnosis of any psychological disorder—Diagnóstico de cualquier trastorno psicológico		
Assunzione di sostanze psicoattive—Psychoactive substance intake—Ingesta de sustancias psicoactivas		
Più di 3 assunzioni di alcool durante la settimana—More than 3 instances of alcohol consumption during the week—Mas que 3 ingestas de alcohol durante la semana		
Condizione di fumatrice—Smoker—Fumo de cigarros		
Singolo abuso sessuale da parte del partner durante l’età adulta (>18 anni)—Single sexual abuse by your partner at adult age (>18 years)—Abuso sexual individual por parte de la pareja durante su edad adulta (>18 años)		
Ricorrenti abusi sessuali da parte del partner durante l’età adulta (>18 anni)—Recurrent sexual abuse by your partner at adult age (>18 years)—Abuso sexual recurrente por parte de la pareja durante su edad adulta (>18 años)		
Singolo abuso fisico da parte del partner durante l’età adulta (>18 anni)—Single physical abuse by your partner at adult age (>18 years)—Abuso físico único por parte de la pareja durante su edad adulta (>18 años)		
Ricorrenti abusi fisici da parte del partner durante l’età adulta (>18 anni)—Recurrent physical abuse by your partner at adult age (>18 years)—Abuso físico recurrente por parte de la pareja durante el embarazo (>18 años)		
Singolo abuso psicologico da parte del partner durante l’età adulta (>18 anni)—Single psychological abuse by your partner at adult age (>18 years)—Maltrato psicológico único por parte de la pareja durante su edad adulta (>18 años)		
Ricorrenti abusi psicologici da parte del partner durante l’età adulta (>18 anni)—Recurrent psychological abuse by your partner at adult age (>18 years)—Abuso psicológico recurrente por parte de la pareja durante su edad adulta (>18 años)		
Singolo abuso sessuale durante l’infanzia (<18 anni)—Single sexual abuse during childhood (<18 years)—Abuso sexual único durante infancia (<18 años)		
Ricorrenti abusi sessuali durante l’infanzia (<18 anni)—Recurrent sexual abuse during childhood (<18 years)—Abuso sexual recurrente durante infancia (<18 años)		
Singolo abuso fisico durante l’infanzia (<18 anni)—Single physical abuse during childhood (<18 years)—Abuso físico único durante infancia (<18 años)		
Ricorrenti abusi fisici durante l’infanzia (<18 anni)—Recurrent physical abuse during childhood (<18 years)—Abuso físico recurrente durante infancia (<18 años)		
Singolo abuso psicologico durante l’infanzia (<18 anni)—Single psychological abuse during childhood (<18 years)—Maltrato psicológico único durante infancia (<18 años)		
Ricorrenti abusi psicologici durante l’infanzia (<18 anni)—Recurrent psychological abuse during childhood (<18 years)—Abuso psicológico recurrente durante infancia (<18 años)		
Esperienze traumatiche durante l’infanzia subite o assistite (lutti, incidenti, violenza assistita, gravi malattie, guerra)—Traumatic experiences during childhood endured or witnessed (bereavement, accidents, assisted violence, serious illnesses, war)—Experiencias traumáticas durante infancia sufrida o presenciada (duelo, accidentes, violencia presenciada, enfermedades graves, guerra)		
Esperienze traumatiche durante l’età adulta subite o assistite (lutti, incidenti, violenza, gravi malattie, guerra)—Traumatic experiences in adulthood endured or witnessed (bereavement, accidents, violence, serious illnesses, war)—Experiencias traumáticas durante edad adulta sufrido o presenciado en la edad adulta (muerte, accidentes, violencia presenciada, enfermedades graves, guerra)		
Patologie psichiatriche in famiglia (madre, padre, zii, nonni)—Psychiatric diseases in the family (mother, father, uncles, grandparents)—Patologías psiquiátricas en la familia (madre, padre, tíos, abuelos)		
Rapporti tesi all’interno della famiglia di origine—Tense relationships within the family of origin—Relaciones tensas dentro de la familia de origen		
Rapporti tesi all’interno dell’attuale nucleo familiare—Tense relationships within the current family unit—Relaciones tensas en todo el interior de la unidad familiar actual		
Buon rapporto con i genitori—Good relationship with parents—Buena relación con los padres		
Storia di aborto/i spontaneo/i—History of spontaneous abortion(s)—Historial de abortos espontáneos		
Storia di interruzione volontaria di gravidanza—History of voluntary termination of pregnancy—Historia de interrupción voluntaria del embarazo		
Soddisfazione per la propria vita così come è—Satisfaction for one’s life as it is—Satisfacción con la vida tal como es		
Elevati livelli di stress—High stress levels—Altos niveles de estrés		
Ricorrenti pensieri suicidari—Recurrent suicidal thoughts—Pensamientos suicidas recurrentes		
Ricorrenti sentimenti di colpa e vergogna—Recurring feelings of guilt and shame—Sentimientos recurrentes de culpa y vergüenza		
Buona autostima—Good self-esteem—Buena autoestima		
Rapporti sessuali soddisfacenti—Satisfactory sexual intercourse—Relaciones sexuales satisfactorias		
Desiderio sessuale—Sexual desire—Deseo sexual		
Amicizie significative—Meaningful friendships—Amistades significativas		
Ottenimento di supporto in caso di bisogno—Getting support in case of need—Obtener apoyo cuando sea necesario		
Impedimento nello svolgimento di normali attività quotidiane a causa di dolore—Impediment in carrying out normal daily activities due to pain—Impedimento para realizar actividades normales adiariamente debido al dolor		
Attività fisica/sport regolare—Regular physical activity/sport—Actividad física/deporte regular		
Stanchezza/assenza di energie anche di prima mattina—Tiredness/absence of energy even in the early morning—Cansancio/falta de energía incluso temprano en la mañana		
Elevata emotività (espressione ed esperienza degli stati emotivi molto intensa)—High emotionality (very intense expression and experience of emotional states)—Alta emocionalidada (expresión y experiencia muy intensa de estados emocionales)		
Più di 4 partner sessuali durante il corso della vita—More than 4 sexual partners during the course of life—Mas que 4 parejas sexuales a lo largo de la vida		
Età del primo rapporto sessuale inferiore a 14 anni—Age of first sexual intercourse less than 14 years—Primera relación sexual antes de los 14 años		
Presenza di dolore durante il rapporto sessuale—Presence of pain during sexual intercourse—Presencia de dolor durante las relaciones sexuales		

## Data Availability

The data supporting the findings of this study have yet to be available as they are part of a research protocol. The data will be available upon reasonable request from the corresponding author upon publication.
